# Elongated Styloid Process Evaluation Using Panoramic Radiography in Patients With Oral Submucous Fibrosis: A Retrospective Study

**DOI:** 10.7759/cureus.60781

**Published:** 2024-05-21

**Authors:** P Jency Evanjelin, Umamaheswari TN

**Affiliations:** 1 Oral Medicine and Radiology, Saveetha Dental College and Hospitals, Saveetha Institute of Medical and Technical Sciences, Saveetha University, Chennai, IND

**Keywords:** retrospective study, radiographic assessment, panoramic radiography, oral submucous fibrosis, styloid process

## Abstract

Background

Oral submucous fibrosis (OSMF) is a chronic condition primarily affecting the buccal mucosa, characterized by fibrotic changes, scarring, and precancerous lesions. Pathologically, it involves inflammation, collagen deposition, and muscular degeneration.

Objective

This study aimed to assess the prevalence and distribution of elongated styloid process (ESP) in patients diagnosed with OSMF, contributing to the understanding of anatomical variations in this population.

Methods

A retrospective study was conducted at the Department of Oral Medicine and Radiology of Saveetha Dental College. We collected records of OSMF patients from 2021 to 2023, which included panoramic radiographs. We evaluated the presence, type, and grading of ESP alongside OSMF grades using standardized classifications.

Results

Out of 125 OSMF patients, 67 (53.6%) had ESP. Type I ESP was most prevalent (83.58%). Bilateral occurrences were more common (52.2%) than unilateral (41.79%). On both sides, type I ESP predominated significantly. Among unilateral cases, left-side occurrences were slightly more frequent. Type I ESP remained predominant regardless of laterality.

Conclusion

The study highlights the high prevalence of ESP, predominantly type I, in OSMF patients, with bilateral occurrences more common. These findings provide valuable insights into the anatomical variations associated with OSMF, contributing to clinical understanding and potential future research directions.

## Introduction

Oral submucous fibrosis (OSMF) is a chronic illness mostly affecting the buccal mucosa that is characterized by the formation of scars, tissue fibrosis, and precancerous lesions [1]. Deep connective tissue or lamina propria localized inflammation, excessive collagen deposition underneath the oral mucosal epithelium, and degenerative changes in the muscles are some of the most important signs of the disease [2]. The prevalence of OSMF varies by ethnicity and geography and is strongly associated with dietary and cultural patterns. Although the illness is most common in India, it is also common in Taiwan, other Asian countries, and South Africa, especially among Indian immigrants [3]. According to reports, trismus accounts for 37.2-90.8% of the patient's clinical presentations; however, 14.2-25.9% of patients also exhibit other symptoms, such as burning sensations, recurring ulcerations, and excessive salivation. The originally soft and pink oral mucosa becomes somewhat pale and inelastic in cases of OSMF [4]. Afterward, the mucosa exhibits a notable decrease in flexibility and opaqueness, accompanied by white blanching. It feels rough and papery white to the touch, and there is a strong vertical band directly across from the premolar region. People who suffer from OSMF frequently feel their mouths burning quite badly after eating spicy food [5]. Later stages also include the lips and palate, with lesions appearing on one or more of these areas. The disease poses a substantial risk of mortality due to its high rate of malignant transformation (1.5-15%) [6].

Autoimmunity, spicy foods, chewing betel nuts, and low levels of vitamins B and C and iron are among the causes of OSMF. Chewing betel nuts is one of the main risk factors for OSMF, according to epidemiological data [7]. According to the World Health Organization (WHO), there are over 5 million OSMF patients worldwide. In India, OSMF occurs more frequently in women, whereas in other regions, the opposite holds true [8]. OSMF has sparked concern among young adults, particularly males, due to the widespread availability of commercially processed areca nut and tobacco products. Tax enforcement of tobacco production regulations, easy availability, attractive packaging, deceptive advertising, and cultural practices are responsible for the rise in OSMF cases. Alkaloids and flavonoids spread when the tobacco mixture is in contact with the oral mucosa for a long time, which causes chemical and mechanical irritation [9]. Ho et al. suggested that the areca nut tract may regulate apoptotic pathways in neutrophils and may compromise the periodontal health of areca nut chewers [10]. As a result, inflammatory cells infiltrate, triggering the production of growth factors such as transforming growth factor-beta (TGF-b), interferon alpha, and tumor necrosis factor. As the disease progresses to a moderate stage, trismus becomes irreversible. This necessitates immediate medical and surgical intervention, followed by rigorous postoperative physiotherapy [11].

Because OSMF does not have a well-established treatment plan, managing it can be quite difficult for clinicians. Fundamentally, the goals of treating OSMF are to reduce the likelihood of malignant transformation, halt the disease's progression, and improve the disease's symptoms. Treatment for OSMF involves a wide range of pharmaceuticals, such as anti-inflammatory drugs like corticosteroids, placental extracts, hyaluronidase, anti-cytokines, vasodilators, and immunomodulators, along with dietary supplements like vitamins and antioxidants. You can inject these methods submucosally, use them topically, or take them orally. Advanced cases of OSMF receive surgical treatments. When managing individuals with OSMF, physical therapy can be utilized alone or in conjunction with other treatment plans. Recurrence is likely caused by ineffective physiotherapy, which manifests as a patient's lack of enthusiasm or pain during physiotherapy exercises that prevents them from engaging in active mouth-opening activities, in addition to the habit's persistence. In some cases of OSMF where a full clinical exam was not possible because of trismus, a similar analysis showed the presence of an elongated styloid process (ESP). These cases involved regular radiographic examinations in the form of orthopantomograms. The styloid process is a slender, elongated bony projection that extends downwards, forwards, and slightly medially from the temporal bone. It emerges from the temporal bone in close proximity to the anteromedial aspect of the stylomastoid foramen. Positioned between the internal and external carotid arteries as well as the internal jugular vein, it typically maintains a straight alignment but may occasionally exhibit curvature. The ESP is considered to be the result of ossification of the styloid process or the stylohyoid ligament. The normal length of the styloid process is 20-30 mm and if it is above 30 mm, it is considered elongated. An otolaryngologist frequently discovers ESP when a patient complains of throat pain. This study aimed to assess the prevalence of ESP in patients with OSMF. The study also aimed to assess the distribution of ESP types, occurrences, and grades of OSMF in the study samples.

## Materials and methods

This retrospective study was conducted in the Department of Oral Medicine and Radiology at Saveetha Dental College. Ethical approval was obtained from the Institutional Human Ethical Committee, Saveetha University (IHEC/SDC/OMED-2101/23/064). The patient’s records of mouth opening and histopathologically proven OSMF cases were collected for the study. Panoramic radiographs taken in 12 seconds at 70 kV and 10 mA using a standard technique were the prerequisites for inclusion. We excluded patients who did not have their panoramic radiographs completed at our radiology department to address the magnification and various factors employed by variable systems. We used a uniform magnification of 1:1.2 throughout. Hospital records of OSMF patients who reported to the department from 2021 to 2023 were collected for the study. Their mouth-opening and histopathological records were also collected for the study. The mouth opening was graded as grade I-IV, according to Ranganathan et al. (2001) [12]. Also, histopathologically, it was graded from I to IV [13].

**Figure 1:**
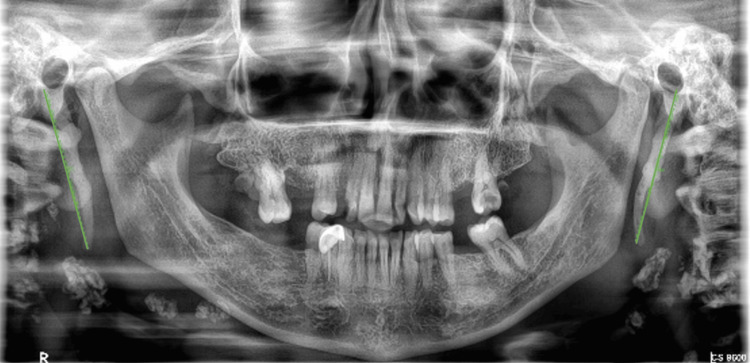
Panoramic image showing the elongated styloid process in a patient with oral submucous fibrosis.

The panoramic radiographs were taken from the Dental Information Archiving Software (DIAS) system presented exclusively to the college. Carestream 9600 (Carestream Dental, Atlanta, GA) used standard exposure parameters to capture the images. The sample size of 125 was determined using G*Power software, based on a power of 95 and an alpha error level of 0.05, as indicated in the seminal work by Shivakumar et al. (2014) [14]. A total of 1250 radiographs were recruited, of which 625 belonged to patients with OSMF. Other 625 were considered as a control group where patients did not have OSMF. Systematic sampling was done to recruit the calculated sample size with a regular block interval size of 5. We found the lengths of the processes on the panoramic X-ray using the software's measurement points from where the process started to where it ended (Figure 1). This was done regardless of whether the styloid process was segmented or not using Langlais classification of elongated stylohyoid ligament complex (1986) [15].

**Figure 2:**
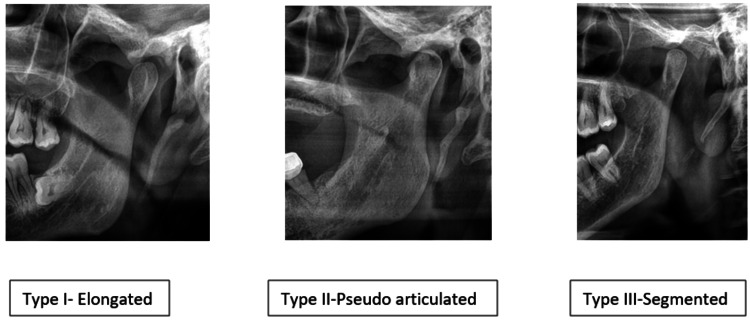
Elongation of styloid process in OSMF patients according to Langlais classification in OPG images. OSMF: oral submucous fibrosis; OPG: orthopantomogram.

We identified significant findings in the styloid process and/or the ossification of the stylomandibular ligament if the length exceeded 25 mm. The Langlais classification is utilized for grading and evaluating the styloid process that has been elongated [15]. According to Langlais classification, type 1 - elongated, type 2 - pseudoarticulated, and type 3 - segmented are considered in the classification (Figure 2). The presence of the elongated styloid process, the side of the elongation, if unilateral or bilateral, and the grading of OSMF and the elongated styloid process were collected and tabulated in a Microsoft Excel sheet (Microsoft Corporation, Redmond, WA). The data were analyzed using SPSS software version 24.0 (IBM Corp., Armonk, NY). The study expressed all the variables in frequency and percentages. A chi-square test was used to assess the relationship between the variables with a significance of less than 0.05.

## Results

The study comprised 125 OSMF patients and 125 in the control group with their panoramic radiographs. The study assessed the grade of OSMF the patients had, the prevalence of ESP, and the pattern and distribution of the same. Out of 125 patients diagnosed with OSMF, 67 (53.6%) patients were identified with an ESP under Langlais classification. In the control group, out of 125 patients, 46 (36.8%) patients had the presence of an ESP. Comparing the OSMF and control group, the OSMF group had a significantly high prevalence of ESP (Table 1).

**Table 1:** Prevalence of elongated styloid process in the OSMF and control group revealing significant differences. OSMF: oral submucous fibrosis. * Statistically significant.

Presence of the styloid process	Patients with OSMF		Control group		P-value
	Present	Absent	Present	Absent	
	67 (53.6%)	58 (46.4)	46 (36.8)	79 (63.2)	0.02*

**Figure 3:**
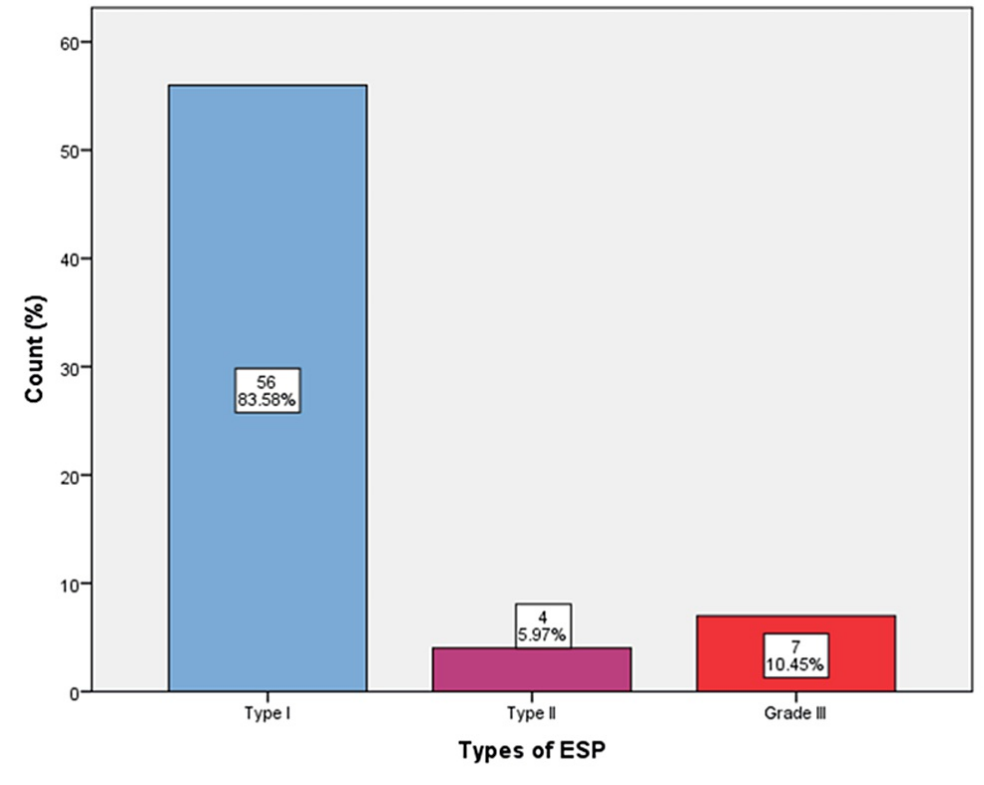
Distribution of elongated styloid process (ESP) among the study samples.

Out of 67 patients who were identified with ESP, 56 (83.58%) had type I, four (5.97%) had type II, and seven (10.45%) patients had type III ESP (Figure 3).

**Figure 4:**
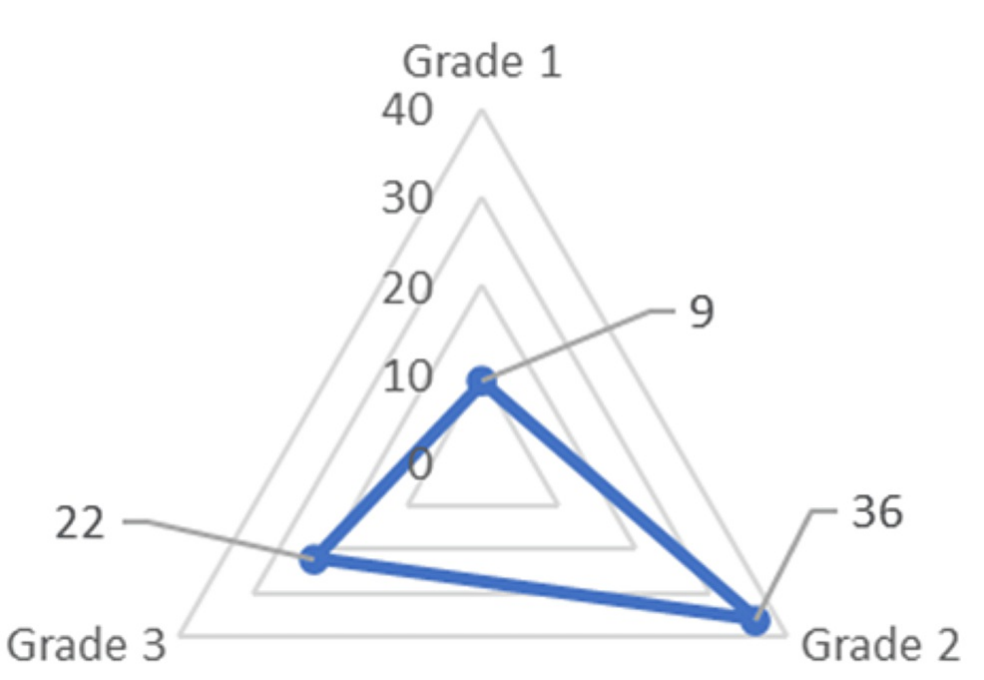
Distribution of grades of oral submucous fibrosis among patients with elongated styloid process using radar graph.

**Figure 5:**
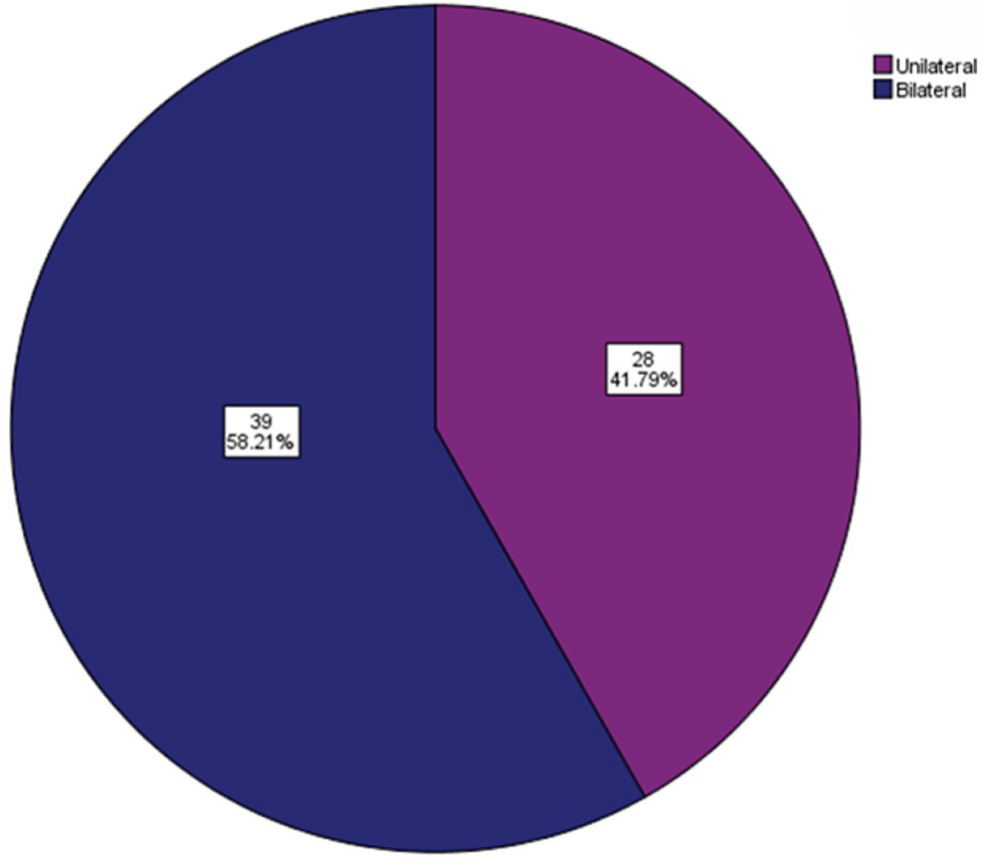
Distribution of type of occurrence of the elongated styloid process.

For the patients with ESP, OSMF was assessed for its grading under classification by Ranganathan et al. [12] and Pindborg et al. [13]. It was revealed that nine (13.5%) patients had grade I, 36 (53.7%) had grade II, and 22 (32.8%) patients had grade III OSMF (Figure 4). The occurrence of ESP was classified into two categories depending on whether it was unilateral or bilateral. In the present study, results revealed that out of the 67 patients with ESP, 28 (41.79%) had unilateral, and 39 (52.2%) patients had bilateral occurrences (Figure 5).

**Figure 6:**
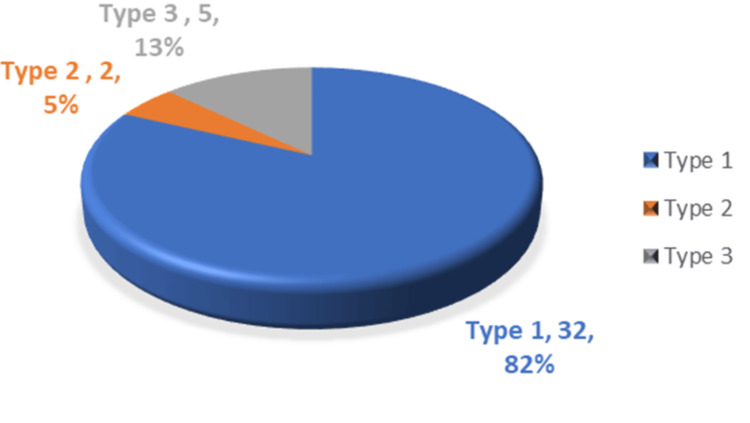
Distribution of the elongated styloid process in bilateral occurrence.

**Figure 7:**
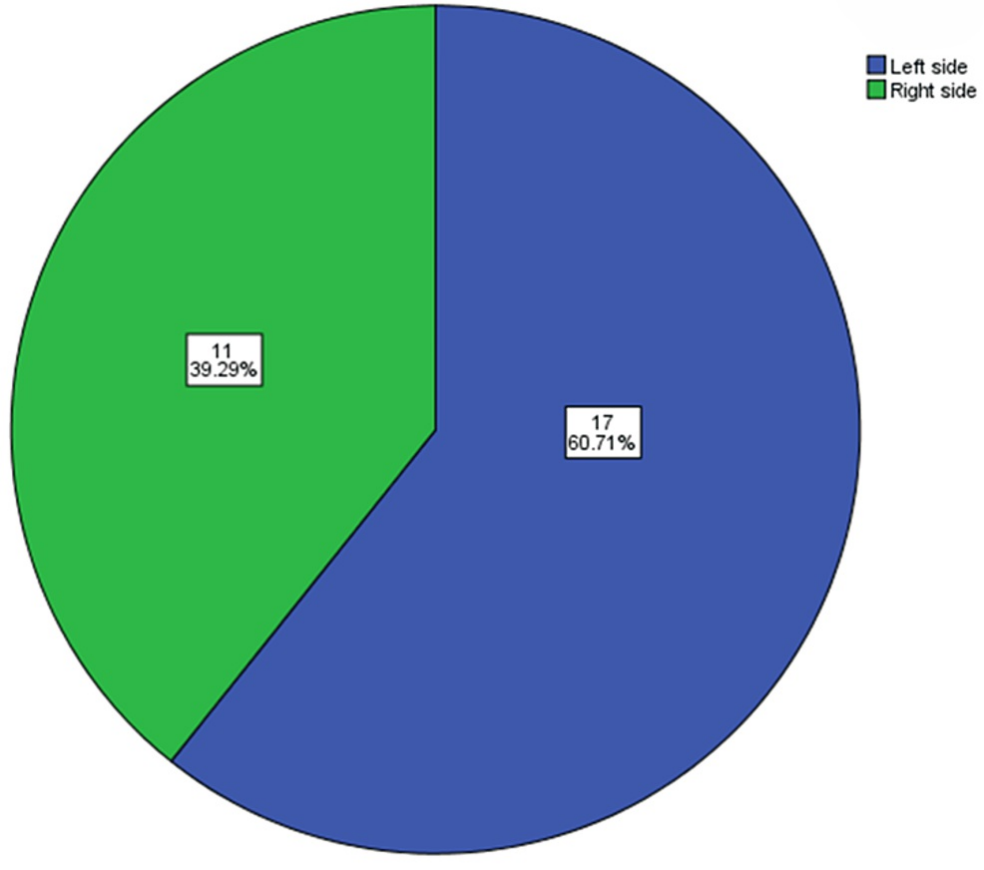
Distribution of side of occurrence in unilateral elongated styloid process patients.

In patients with bilateral occurrence, 32 (82%) had type I, two (5%) had type II, and five (13%) had type III ESP (Figure 6). Patients with unilateral occurrences were again divided into groups based on the side of the occurrence. Out of the 28 patients with unilateral ESP, 17 (60.71%) had occurrences on the left side and 11 (39.29%) had occurrences on the right side (Figure 7).

**Figure 8:**
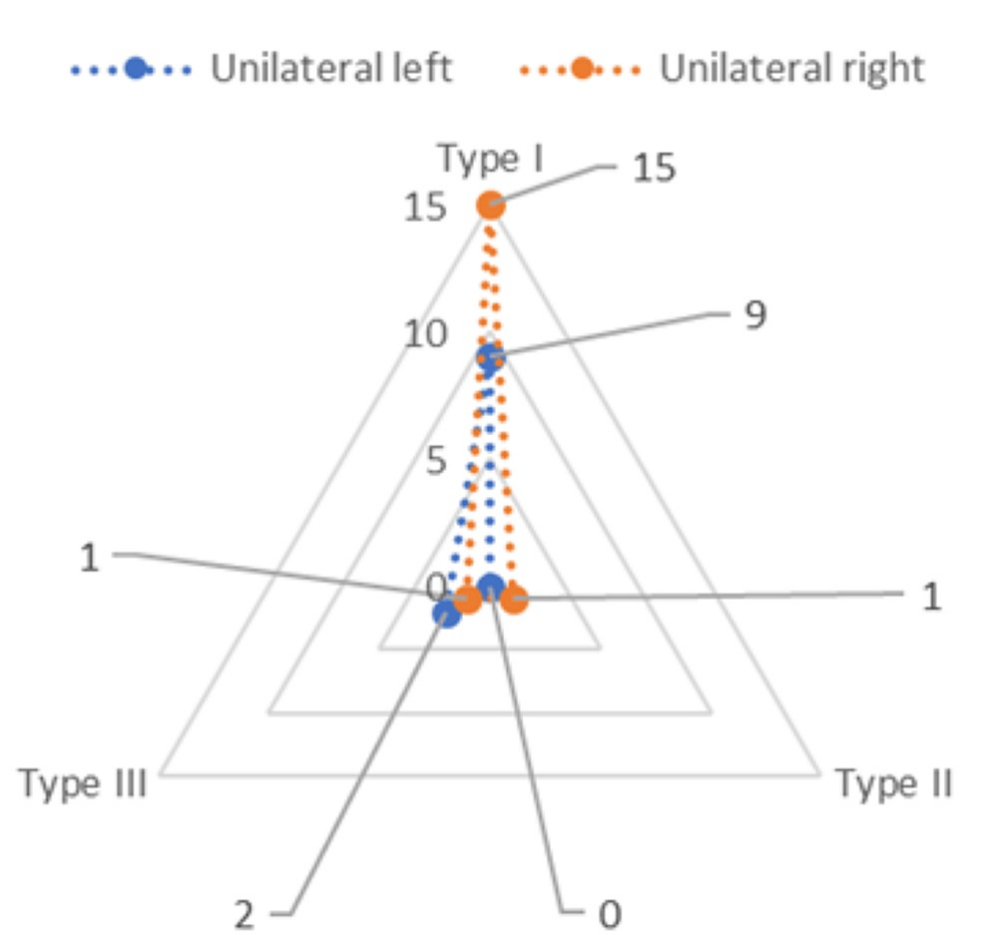
Distribution of type of elongated styloid process in the left and right unilateral occurrence.

On the right side, nine (81.82%) patients had type I, and two (18.18%) patients had type III ESP, whereas on the left side, 15 (88.24%) patients had type I, one (5.88%) patient had type II, and one (5.88%) patient had type III ESP (Figure 8). This distribution had predominantly type I ESP, and it had a significantly higher prevalence on both the left and right sides (Table 2).

**Table 2:** Distribution of different types of elongated styloid process in patients with oral submucous fibrosis. * Statistically significant.

Type of elongation	Bilateral, n (%)	Left unilateral, n (%)	Right unilateral, n (%)
Type I	32 (82)	15 (88.24)	9 (81.82)
Type II	2 (5)	1 (5.88)	0
Type III	7 (13)	1 (5.88)	2 (18.18)
P-value		0.042*	

## Discussion

Stylos, meaning "pillar" in Greek, is the origin of the phrase "styloid process" (SP). This structure is a long, spherical, cartilaginous bone that is on the lower part of the temporal bone. It is next to the carotid canal and jugular foramen, behind the mastoid apex, and anterior to the stylomastoid foramen. On the medial side of the SP are the internal jugular vein and the cranial nerves VII, IX, X, XI, and XII. Laterally, the SP tip is near the external carotid artery; medially, it is near the internal carotid artery and its supporting sympathetic chain. It develops in conjunction with the stylohyoid apparatus, the stylohyoid ligament, and a small hyoid bone horn. The SP gives rise to the styloglossus, stylohyoid, and stylopharyngeus muscles. The styloid and stylomandibular ligaments also link to the SP [16]. Both unilateral and bilateral elongation of the SP are possible, although unilateral elongation occurs more frequently. The prevalence of stylohyoid ligament ossification varies; it can range from 2.4% to 84.4%, with no apparent symptoms [17].

There are reports that the styloid process can vary in length from 15.2 mm to 47.7 mm. Numerous researchers have noted that the styloid process often measures 20 to 30 mm in length, with 31 mm and above considered elongated [18]. Congenital extension of the styloid process, calcification of the stylohyoid ligament, and the development of osseous tissue at the ligament's insert are all possible explanations for the styloid process's elongation; however, the exact origin remains a mystery [19]. Clinical palpation of this structure in the ipsilateral tonsillar fossa can identify the elongation of the styloid process. However, since most of the patients have no symptoms, the panoramic radiograph, which is often obtained for different purposes, often shows the styloid process to be elongated. People commonly employ the panoramic radiograph to detect elongation of the styloid process. However, other radiographic views, such as the mandible's posterior-anterior projection, lateral oblique projection, axial, and cephalometric radiographs, can also provide valuable information for assessing the elongated process. Scaf et al. deem the styloid process to have mineralization if its length exceeds 30 mm and it extends beyond the temporal bone on panoramic radiographs [20].

In this study, a panoramic radiograph was also used to assess the grading of OSMF and ESP. In the present study, type I ESP was more predominant than other types on both the left and right sides, and it was statistically significant. Similarly, in other studies, type I ESP has been concluded to be predominant [21]. Also, in the present study, the prevalence of left unilateral ESP was higher than on the right side. Many studies have found similar results. We can attribute this difference to long-term one-sided activity, suggesting a potential role of chewing in the hardening of the styloid process [22]. In the present study, bilateral occurrences were more common than unilateral occurrences. Some studies have found similar results [23]. Importantly, in the present study, there is a predominance of ESP present among OSMF patients. Similarly, Patel et al. concluded in their case report that ESP can be a restricting factor in treating OSMF patients, and Shivakumar et al. also concluded that ESP can influence progressive OSMF [24]. The clinical manifestations of stylalgia, which is the pain experienced by patients due to ESP, frequently result in the patient experiencing excruciating pain and suffering because of incorrect diagnoses and unsuccessful prior treatments, even from skilled medical professionals. This is due to the fact that patients frequently report headaches, otalgia, dysphagia, dull, persistent, recurrent pain in the oropharynx, and pain during neck rotation [25]. Conditions that irritate the sympathetic nervous system, such as temporomandibular joint dysfunctions, muscular inflammation, otitis media, pharyngeal infections, and chronic degenerative and inflammatory conditions, frequently overlap with these symptoms, which are frequently non-specific in their location or origin [26]. This suggests that to determine an accurate diagnosis and appropriate course of treatment, careful clinical examination supported by radiological results must be given the utmost emphasis [27]. When trismus prevents a complete clinical assessment, an accompanying extended styloid process may be visible on routine radiographic exams, such as an orthopantomogram.

Neonatal origins of the styloid process later mineralized, particularly the ceratohyal portion that forms the stylohyoid ligament; symptoms include otalgia, headaches, dizziness, and nebulous facial discomfort during swallowing and head movement [28]. The potential activation of residual Reichert's cartilage in the styloid complex, which results in ongoing ossification in adulthood as a consequence of sustained trauma-induced TGF-β production and stimulation of osteoprogenitor cells, is indicated by the involvement of TGF-β in collagen deposition and chondrocyte maturation [26].

The present study has many advantages. It has a huge sample, and it has assessed the underlying mechanism. However, the retrospective study design has a significant limitation. Also, without OSMF patients, there was no control group for assessing styloid process elongation. There was also a bias in radiographic quality, like the wrong magnification, equipment error, and patient handling while taking the radiograph.

## Conclusions

In conclusion, this retrospective study focused on assessing the radiographs of OSMF patients, shedding light on the prevalence and distribution of ESP among this specific population. The results reveal a significant prevalence of type I ESP on both the left and right sides. Remarkably, type I ESP emerged as the most common subtype on both sides, demonstrating statistical significance. Furthermore, the study revealed that bilateral occurrences of ESP were more prevalent than unilateral occurrences, emphasizing the bilateral nature of this anatomical variation in OSMF patients. The observed prevalence and distribution patterns align with similar findings reported in other studies, consolidating the notion of a consistent association between OSMF and ESP. Notably, the study contributes to the existing body of knowledge by reaffirming the predominance of ESP in OSMF patients. Additionally, the statistical significance of these findings adds weight to the understanding of the anatomical variations in this specific patient population. The insights gained from this retrospective analysis may prove valuable in guiding future research and clinical considerations related to OSMF and ESP. Further investigations are needed to unveil the underlying cause of this relationship.
